# Age-Related Differences Across Adulthood in IMU-Derived Gait Quality During Habitual Walking

**DOI:** 10.3390/s26072194

**Published:** 2026-04-02

**Authors:** Jiahui Wang, Abner Sergooris, Annick A. A. Timmermans, Benedicte Vanwanseele

**Affiliations:** 1 Human Movement Biomechanics Research Group, Department of Movement Sciences, KU Leuven, 3001 Leuven, Belgium; benedicte.vanwanseele@kuleuven.be; 2 Faculty of Rehabilitation Sciences, Hasselt University, 3590 Diepenbeek, Belgium; abner.sergooris@uhasselt.be (A.S.); annick.timmermans@uhasselt.be (A.A.A.T.)

**Keywords:** healthy ageing, gait quality, inertial measurement unit (IMU), gait biomechanics, gait symmetry, local dynamic stability, smoothness, habitual walking, hip muscle strength, wearable sensors

## Abstract

**Highlights:**

**What are the main findings?**

- Step symmetry and stability during habitual walking declined with age.
- Gait quality was more strongly associated with age than with physical activity.
- Hip muscle strength showed selective relationships with gait quality beyond ageing effects.

**What are the implications of the main findings?**

- Single lower-back IMU analysis during habitual walking captures subtle ageing-related gait quality changes.
- Hip muscle strength may contribute further to specific gait quality characteristics.

**Abstract:**

Age-related changes in walking are often evaluated using performance-based measures, but little is known about how trunk-derived gait quality changes across healthy adulthood during habitual walking. This study examined gait quality using a single inertial measurement unit positioned at the lower back to record acceleration and angular velocity signals during approximately 5 min of continuous self-selected overground habitual walking in healthy adults across multiple age groups spanning adulthood. Step and stride symmetry were derived from trunk acceleration autocorrelation, local dynamic stability was quantified using the maximum Lyapunov exponent, and smoothness was derived from trunk angular velocity. Associations with age were evaluated, and additional analyses examined whether hip muscle strength and physical activity contributed to inter-individual variation in these gait measures. Age was associated with lower step symmetry and reduced local dynamic stability, whereas smoothness showed more limited age-related changes. Hip extensor and internal rotator strength explained additional variance in specific gait quality measures, while physical activity showed limited associations. These findings indicate that a single lower-back IMU can detect subtle age-related differences in interpretable gait quality during habitual walking across adulthood.

## 1. Introduction

Ageing is associated with progressive changes in walking and contributes substantially to mobility limitations, fall risk, and reduced independence across adulthood [1,2]. Traditional clinical assessments, such as walking speed or functional mobility tests, provide valuable information regarding performance capacity but offer limited insight into the underlying biomechanical characteristics of gait. Individuals with similar performances on these tests may still differ in gait symmetry, local dynamic stability, or smoothness. Laboratory-based motion analysis systems enable detailed biomechanical evaluation of gait kinematics and kinetics; however, their reliance on specialized equipment and controlled environments may limit broader clinical and ambulatory application. Inertial measurement units (IMUs) have therefore emerged as an alternative approach for assessing signal-based gait biomechanics under ambulatory settings, particularly when continuous walking recordings are required [3].

Early studies showed that trunk-mounted accelerometers and gyroscopes can capture whole-body movement patterns during walking and provide clinically relevant information on gait cycle characteristics. In particular, a single sensor positioned at the lower back captures tri-axial acceleration and angular velocity signals reflecting overall gait behaviour, from which interpretable gait characteristics such as symmetry, local dynamic stability, and smoothness can be derived [4,5,6]. Lower-back IMU analysis has been applied across older and clinical populations, demonstrating sensitivity to subtle differences in gait that may not be captured conventional performance-based measures alone [7,8,9].

Despite increasing use of lower-back IMU analysis, findings regarding age-related differences in trunk-derived gait quality across healthy adulthood remain incomplete. Some studies report reduced symmetry or altered stability with advancing age, whereas others observe minimal or axis-specific differences during controlled short laboratory walking protocols [7,8]. One likely reason for these mixed findings is methodological heterogeneity across studies, including differences in age grouping, walking duration, and the specific gait characteristics examined. In particular, many investigations have relied on short controlled walking trials, which may limit the characterization of subtle age-related differences in trunk-derived gait behaviour. Longer continuous walking protocols may therefore provide a more robust assessment of gait-quality measures by capturing a larger and more standardized number of strides [10,11].

Beyond chronological age, inter-individual differences in neuromuscular capacity and lifestyle may also contribute to variability in gait biomechanics across adulthood. Hip muscle strength plays a central biomechanical role in walking through its contribution to body support, propulsion, and balance, and ageing-related shifts in joint kinetics suggest increased reliance on hip musculature during locomotion [12,13]. Inter-individual differences in hip muscle strength may therefore influence the extent to which this proximal reliance contributes to gait variability across adulthood. Physical activity represents a lifestyle-related characteristic that may influence locomotor behaviour and physical capacity; however, relationships between self-reported activity and detailed biomechanical gait characteristics remain incompletely understood [14,15]. Considering these factors alongside chronological age may therefore provide a more comprehensive understanding of gait quality across adulthood.

Related studies, including previous work in clinical populations, have shown that trunk-IMU-derived gait-quality measures can capture meaningful locomotor differences [16]. Studies in healthy and older adults have also identified age-related differences in selected trunk-derived gait characteristics, including symmetry, regularity, and local dynamic stability [17,18]. However, previous work has more often examined isolated outcomes, younger-versus-older contrasts, treadmill protocols, or shorter walking assessments, rather than providing a broader reference framework across adulthood using multiple interpretable trunk-derived gait-quality measures. Consequently, healthy adulthood-spanning reference patterns across these measures remain less clearly characterized. Therefore, the present study aimed to examine age-related differences in trunk-derived gait quality across adulthood using a single lower-back IMU during a continuous self-selected overground walking protocol (approximately 5 min). A secondary aim was to explore whether hip muscle strength and physical activity contribute to age-related differences in gait quality.

## 2. Materials and Methods

### 2.1. Participants

The study was designed to recruit a total of 90 healthy adults following approval from the Social and Societal Ethics Committee (SMEC) of KU Leuven (G-2024-8735), with 15 participants in each decade of age from the 20s to the 70s to ensure balanced age stratification across the adult lifespan. Eligibility criteria included independent walking ability over at least 10 m, and the capacity to ascend and descend stairs, ensuring that participants could complete all locomotor tasks included in the assessment protocol. Exclusion criteria comprised current or recent lower-limb pain or injury, major cardiovascular or systemic disease (e.g., uncontrolled hypertension or heart failure), recent orthopedic surgery involving the lower extremities, substantial mobility limitations, or cognitive impairment. Test–retest reproducibility of the protocol has been reported previously [16]. All assessments were conducted in an indoor sports facility (Building De Nayer, Leuven, Belgium).

### 2.2. Experimental Protocols

#### 2.2.1. Gait Quality Assessment

Gait quality was assessed during continuous, self-selected straight-line walking lasting approximately 5 min. No verbal instructions regarding walking speed or gait behavior were provided. Gait was recorded using a single IMU (Byteflies, Antwerp, Belgium) positioned at the L5/S1 level using a standardized waist belt. The sensor continuously recorded tri-axial accelerations and angular velocities at 200 Hz.

**Figure 1:**
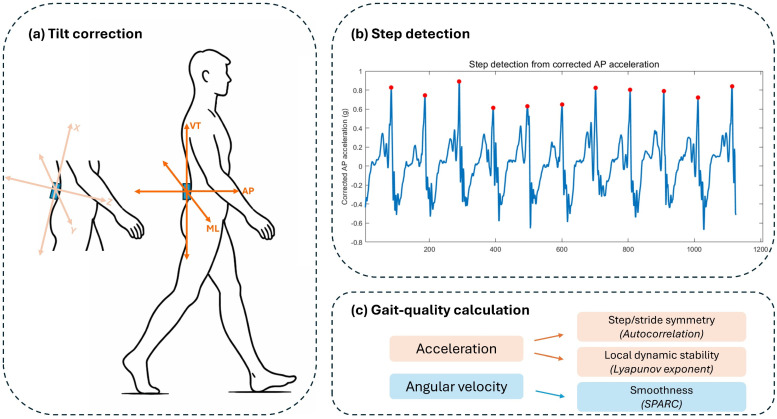
Overview of the gait-signal processing workflow. (**a**) Sensor tilt correction was applied to align raw lower-back IMU signals using global AP, ML, and VT axes. (**b**) Step events were identified from the corrected AP acceleration signal. (**c**) Detected steps were used for step/stride segmentation and for calculating gait-quality parameters. Step and stride symmetry and local dynamic stability were derived from corrected acceleration signals using autocorrelation and the Lyapunov exponent, respectively, whereas smoothness was derived from angular velocity signals using the spectral arc length (SPARC).

Raw sensor signals were reoriented to the global anteroposterior (AP), vertical (VT), and mediolateral (ML) coordinate system by estimating sensor tilt from the gravitational component of the acceleration signal and applying tilt correction following established trunk accelerometry procedures (Figure 1a) [4]. Step events were finally from the AP acceleration signal using a peak-based algorithm and manually checked to confirm detection accuracy (Figure 1b). Detected gait events were visually checked to confirm quality [19]. The shortest recording contained 300 strides; therefore, all recordings were truncated to 300 strides to ensure comparability across participants. Using a fixed number of strides ensured comparability across participants and reduced potential bias related to unequal stride numbers, which is particularly relevant for stability estimation.

Gait quality was characterized using four parameters: step symmetry, stride symmetry, stability, and smoothness, computed according to previously published methods (Figure 1c) [16]. Step and stride symmetry were obtained from the dominant peaks of the unbiased autocorrelation of trunk acceleration, indicating similarity between consecutive steps and strides [5]. Local dynamic stability was quantified using the maximum Lyapunov exponent derived from trunk acceleration [20]. Smoothness was calculated from trunk angular velocity (yaw, pitch, and roll) during walking [21].

All data processing was performed using MATLAB R2024b (MathWorks, Natick, MA, USA).

In addition to gait quality, participant characterization included clinical performance measures, including 40 m fast walking performance (40MFW), the Timed Up and Go test (TUG), stair negotiation, and the 30 s chair stand test (30SCS).

#### 2.2.2. Muscle Strength

Isometric hip muscle strength was evaluated for both limbs using a handheld dynamometer (MicroFet2®, Hoggan Scientific, LLC, Salt Lake City, UT, USA) following standardized testing positions [22]. Strength of the hip abductors (side lying), adductors (supine), extensors (prone), flexors (sitting), internal rotators (sitting) and external rotators (sitting) was assessed. Following a familiarization contraction, two maximal trials were performed for each muscle group, and the highest value was retained for analysis. Two trials were selected to reduce fatigue-related performance decline during repeated maximal contractions while ensuring reliable maximal force measurement.

#### 2.2.3. Physical Activity

Physical activity was assessed using the long, self-administered International Physical Activity Questionnaire (IPAQ), referring to physical activity performed during the previous seven days [14]. Reported frequency (days/week) and duration (minutes/day) of vigorous-intensity, moderate-intensity, and walking activities across work, transportation, household, and leisure domains were converted into metabolic equivalent task minutes per week (MET-min/week) using standard IPAQ scoring procedures (8.0 METs for vigorous activity, 4.0 METs for moderate activity, and 3.3 METs for walking). Total physical activity was calculated as the sum of MET-min/week across all domains. Participants were additionally classified into low, moderate, or high physical activity categories according to established IPAQ cut-offs.

### 2.3. Statistics

Linear regression analyses were conducted with age as a continuous predictor and gender and BMI included as covariates. Quadratic age terms were only retained when they resulted in a statistically significant improvement in model fit. Regression coefficients are reported with standard errors, and model performance was evaluated using the root mean square error (RMSE) and the coefficient of determination (R^2^).

Group differences across age were examined using analysis of covariance (ANCOVA), adjusting for gender and body mass index (BMI) as covariates. For BMI, models were adjusted for gender only. F statistics and corresponding *p* values were obtained for the age-group effect. Effect size was expressed as ω^2^ (omega squared), which quantifies the proportion of variance attributable to the age-group effect, together with 95% confidence intervals estimated using stratified bootstrap resampling. Statistical significance was defined as *p* < 0.05. Post hoc pairwise comparisons were performed when appropriate, with Bonferroni correction for multiple testing.

Multiple linear regression analyses were performed separately for each gait parameter. All models included age (and quadratic age terms when applicable), gender, and BMI as covariates. To examine the additional contribution of physical activity and muscle strength, metabolic equivalent of task (MET), bilateral mean muscle strength of both limbs (MS_average; averaged between left and right within each muscle group), and between-limb muscle strength difference (MS_diff; calculated within each muscle group) were added individually to the base model. MET, MS_average, and MS_diff were not included simultaneously in the same model to avoid multicollinearity as these predictors were statistically interrelated.

All analyses were conducted in R (version 4.4.1) using RStudio (version 2023.6.0.421, RStudio, Inc., Boston, MA, USA).

## 3. Results

A total of 90 healthy adults were recruited for the analysis, with 15 participants per decade from the 20s to the 70s. Subject characteristics are shown in Table 1. After adjustment for gender and body mass index, significant age-group differences were observed for all clinical performance measures, including 40MFW, TUG, stairs, and 30SCS tests. Mean values indicated a general age-related decline in clinical performance, with poorer outcomes observed in the older age groups.

When age was treated as a continuous predictor, significant age-related associations were observed for selected gait quality parameters (Table 2). Significant age effects were identified for step symmetry in the VT and ML directions, as well as a nonlinear association in the AP direction. In addition, gait stability showed significant age-related declines in all three directions (VT, ML, and AP), and smoothness VT exhibited a significant nonlinear relationship with age.

**Figure 2:**
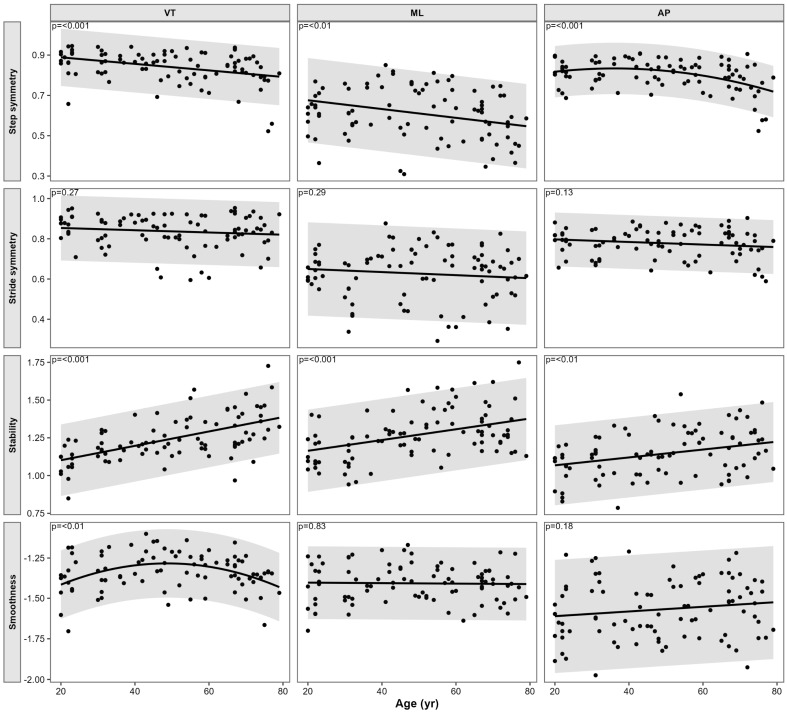
Scatter plots and regression analyses of habitual gait quality parameters across ages. Each panel shows individual observations for step symmetry, stride symmetry, stability, and smoothness derived from trunk inertial signals in the vertical (VT), mediolateral (ML), and anteroposterior (AP) directions. Solid lines indicate fitted regression models with shaded confidence bands. Panels are organised by gait parameter (rows) and movement direction (columns). Higher symmetry values indicate greater gait symmetry. Local dynamic stability was quantified using the maximum Lyapunov exponent; therefore, higher values indicate lower stability. Smoothness was derived from angular velocity, with values closer to zero indicating smoother motion.

As illustrated in Figure 2, these associations were characterized by consistent age-related trends, with step symmetry and stability decreasing across the adult lifespan, indicating progressively reduced gait quality with advancing age.

For gait quality, significant age-group effects were detected for step symmetry AP and stability in all three directions (VT, ML, and AP) (all *p* ≤ 0.05) (Table 3).

**Figure 3:**
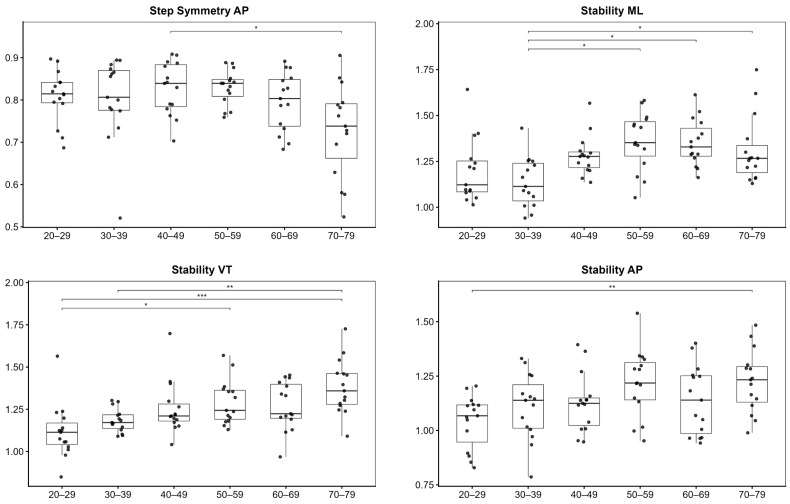
Post hoc age-group comparisons for gait quality parameters are presented for step symmetry AP and stability in the VT, ML, and AP directions. Significance levels are indicated by asterisks: * *p* < 0.05, ** *p* < 0.01, *** *p* < 0.001.

Post hoc analyses (Figure 3) indicated that, for step symmetry AP, significant differences were mainly driven by contrasts between the 70–79-year group and younger age groups, with the most pronounced differences observed relative to the 40–49-year group, whereas differences among younger age groups were less marked.

For stability in all three directions, post hoc analyses showed that age-related differences were primarily attributable to contrasts between younger and older age groups, with the largest differences involving the 60–69- and 70–79-year groups compared with the 20–29- and 30–39-year groups. Overall, stability progressively reduced across a wide age range.

**Figure 4:**
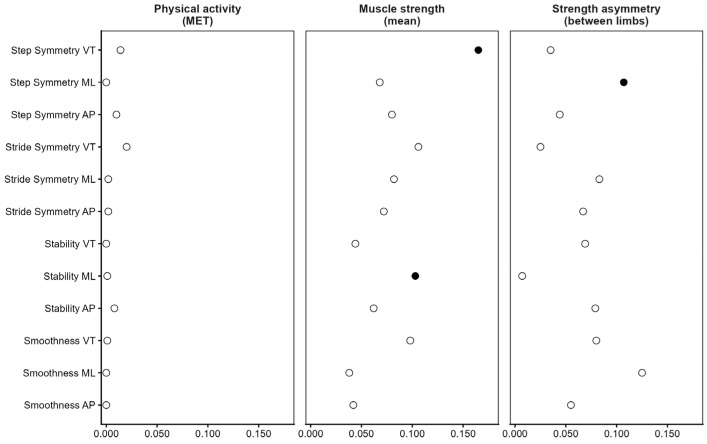
Incremental contribution of physical activity and muscle strength to age-related differences in gait quality. For each gait quality parameter (step symmetry, stride symmetry, stability, and smoothness) and direction (vertical [VT], mediolateral [ML], and anteroposterior [AP]), the additional explained variance (ΔR^2^) is shown when adding physical activity (MET), mean muscle strength, or between-limb strength asymmetry to a base model including age (linear or quadratic, when applicable), gender, and BMI. All panels share the same *x*-axis scale to facilitate comparison across model blocks. Filled circles indicate a significant likelihood-ratio test for the added block (*p* < 0.05); open circles indicate non-significant contributions.

Figure 4 shows the incremental explained variance (ΔR^2^) associated with physical activity and muscle strength when added to a base model including age, gender, and BMI. Physical activity and muscle strength values are provided in Supplementary Tables S1 and S2. The inclusion of physical activity (MET) did not significantly improve model fit for any gait quality parameter across directions, with ΔR^2^ values remaining minimal and all likelihood-ratio tests remaining non-significant. In contrast, mean muscle strength demonstrated parameter- and direction-specific contributions. A significant increase in explained variance was observed for step symmetry in the VT direction and for stability in the ML direction, indicating that overall muscle strength accounted for a small but statistically significant proportion of age-related variance in these gait measures. Between-limb strength asymmetry showed a significant incremental contribution observed only for step symmetry in the ML direction.

**Figure 5:**
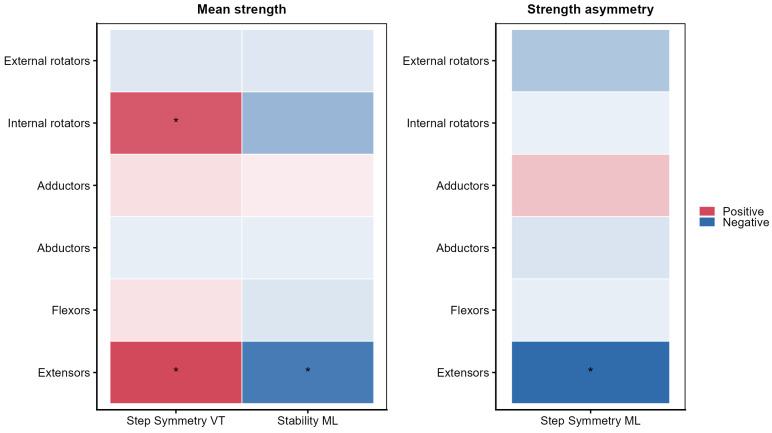
Muscle-group-specific associations with gait quality parameters from regression models, including bilateral mean muscle strength (MS_average) or between-limb muscle strength difference (MS_diff). Red indicates positive associations and blue indicates negative associations; darker shading indicates stronger evidence. Asterisks indicate statistically significant associations (*p* < 0.05).

Figure 5 presents secondary analyses examining muscle-group-specific associations with the gait quality parameters that showed significant incremental effects. For mean muscle strength, positive associations with step symmetry in the VT direction were observed for the internal rotators (*p* = 0.004) and extensors (*p* = 0.002), indicating that greater strength in these muscle groups was associated with higher VT step symmetry. In addition, higher extensor strength was associated with lower Lyapunov exponent values in the ML direction, indicating greater ML gait stability (*p* = 0.004). For between-limb strength asymmetry, a negative association was identified between extensor strength asymmetry and step symmetry in the ML direction (*p* < 0.001), indicating that greater asymmetry in extensor strength was associated with lower ML step symmetry.

## 4. Discussion

Across adulthood, gait quality exhibited age-related differences, with step symmetry and gait stability consistently emerging as the most age-sensitive parameters in all directions. These age-related variations were observed both in age-group comparisons and when age was treated as a continuous predictor, indicating gradual rather than abrupt changes across the adult lifespan. Muscle strength explained a proportion of age-related variances in gait quality beyond age, gender, and BMI, whereas physical activity did not contribute meaningfully to these differences.

We observed an age-related decrease in step symmetry during habitual walking in the VT, ML, and AP directions, with the most distinct decade-level separation occurring in the AP axis (e.g., AP symmetry ~0.80–0.83 from the 20 s–50 s versus ~0.73 in the 70 s). This pattern aligns with previous accelerometry studies reporting lower trunk-acceleration symmetry in older compared with younger adults. For example, Kobayashi et al. demonstrated significantly reduced AP symmetry in older adults, confirming an age-related reduction in gait symmetry [7]. Notably, symmetry values in the present cohort were generally lower than those reported previously. One possible explanation might be the use of a relatively large and controlled number of strides (~300), which may have improved the accuracy of the autocorrelation estimates and enhanced sensitivity to subtle age-related trends that may be less apparent in shorter walking trials, such as those in Kobayashi et al [7]. Similarly, Kobsar et al. found that age-group differences were most evident for AP acceleration measures, suggesting that AP symmetry, reflecting symmetry of forward propulsion generated by both legs, may be particularly sensitive to ageing-related gait changes even during habitual walking [8].

During habitual walking, stability declined progressively across adulthood in both continuous age analyses and age-group comparison analyses, indicating age-related reductions in gait stability. Descriptively, stability values increased from younger to older decades (with higher values indicating lower stability) (VT stability ~1.12–1.18 in early adulthood and ~1.38 in the 70 s), suggesting greater divergence of gait motion with ageing. These findings are consistent with early trunk-accelerometry studies that demonstrated that ageing is associated with increased trunk movement variability and altered gait control patterns [5]. Similarly, nonlinear gait dynamics studies using Lyapunov-based stability metrics have shown that local dynamic stability decreases with age, suggesting reduced gait stability even in healthy adults [23,24]. In contrast, previous investigations using shorter habitual-walking strides or alternative stability definitions have reported mixed or modest age effects, likely due to shorter walking bouts or methodological differences in stability quantification. The present findings extend this body of work by applying trunk IMU-derived Lyapunov stability measures across multiple adult decades during habitual walking, and suggested that wearable sensor-derived stability measures may be sensitive to age-related differences across adulthood [17,25]. In addition, stability metrics are known to depend on data length and signal consistency; methodological studies have shown that the reliability of Lyapunov-based stability estimates generally improves with longer continuous walking periods [26,27]. In this context, the use of approximately 300 controlled strides in the present study may have enhanced estimate robustness and facilitated detection of subtle age-related differences during habitual walking.

Physical activity contributed little additional explanatory value to age-related differences in gait quality in the present analyses. Importantly, physical activity did not decline monotonically with age in this cohort, with relatively high activity levels observed even in older decades, indicating that the observed reductions in gait stability and symmetry occurred despite preserved self-reported physical activity. One potential explanation is methodological: physical activity was assessed using a self-report questionnaire (IPAQ), and systematic reviews have shown low-to-moderate agreement between questionnaire-derived activity estimates and objective sensor-based measurements, with substantial individual-level misclassification that may attenuate associations with IMU-derived gait metrics [28,29,30]. In addition, observational ageing studies suggest that habitual physical activity is often associated with global mobility measures such as gait speed [31,32], whereas age-related differences in gait variability and local dynamic stability have been reported even in high-functioning older adults [25,33]. Therefore, the absence of a strong physical activity effect in the present study should not be interpreted as evidence that activity is unimportant; rather, it suggests that habitual activity alone may be insufficient to fully offset progressive ageing-related changes in trunk-based gait movement during habitual walking.

In the present study, greater hip extensor strength was associated with improved VT step symmetry and enhanced ML stability, whereas larger between-limb differences in hip extensor strength were related to poorer ML symmetry. Given that these gait-quality outcomes were derived from a lower-back sensor positioned near the pelvis, this association may also reflect the contribution of hip muscle function to pelvic control and gait stability during walking. Previous research has linked lower-limb strength, including hip strength, to walking performance and balance across ageing and clinical populations, although most studies have focused on joint-level gait mechanics or clinical performance outcomes rather than trunk-derived gait quality [12,34]. From a biomechanical perspective, hip extensors such as the gluteus maximus contribute to stance support and forward propulsion during walking [35,36], providing a plausible context for the association between greater extensor strength and more symmetrical and stable gait quality observed here. The relationship between hip extensor strength asymmetry and poorer ML symmetry aligns with broader gait frameworks that suggest that bilateral strength imbalance may accompany asymmetrical movement patterns [25,37]. Associations involving hip internal rotator strength are less clearly represented in the existing symmetry literature. Muscles contributing to internal rotation, including the gluteus minimus, anterior gluteus medius, and tensor fasciae latae, also participate hip and pelvic stabilization during single-limb support [38]; however, current evidence does not support a direct muscle-specific pathway for trunk-based symmetry measurement, and this finding should therefore be interpreted cautiously. Taken together, the present results suggest that hip muscle capacity may not only relate to global gait performance but also to trunk-derived symmetry and stability during the continuous walking protocol used in this study, extending previous work that has primarily examined joint-level mechanics or clinical outcomes.

Several limitations should be considered when interpreting the present findings. First, participants were recruited across predefined age decades to facilitate lifespan comparisons. Although the age distribution within some decades was not perfectly uniform, age-related effects were additionally examined using continuous age models, allowing gradual age-related trends to be evaluated beyond categorical grouping alone. Second, although gender distribution across age decades was not perfectly balanced, gender was included as a covariate in all analyses to minimise its potential influence on the reported associations, which is common in observational gait research where demographic balance is not the primary focus. Third, findings should be interpreted within the context of habitual overground walking performed in a standardized setting and the specific gait quality parameters examined. Substantial inter-individual variability may also exist within age groups, and a more complete understanding of participant-level outliers would likely require broader multidomain assessment in future work. The present findings provide baseline reference data for continuous straight overground walking in healthy adults. Building on this foundation, future studies could examine how similar gait-quality measures can be extended to more complex locomotor tasks, such as turning or obstacle negotiation, and to pathological populations, where task-specific analytical and interpretative approaches may be required. Trunk-derived gait metrics can be sensitive to stride number and walking duration, and the extended walking bouts used here may enhance measurement reliability while differing from shorter clinical walking tests [39]. Fourth, despite efforts to recruit broadly across adulthood, the older participants represented a relatively active and healthy cohort, with higher walking and moderate-intensity activity levels than typically reported in population-based ageing studies [40,41]. This likely reflects a common healthy-volunteer effect in lifespan gait research, where individuals willing to participate in extended walking protocols tend to be more active [42]. While this may limit generalizability to less active populations, it also suggests that the observed age-related differences in trunk-derived gait quality were present even under favorable lifestyle conditions and may therefore represent conservative estimates of ageing-related change. In addition, muscle strength assessment was limited to the hip; therefore, the present study does not allow for the evaluation of the relative contribution of other muscle groups to age-related gait adaptations. Finally, methodological choices such as analysis length and preprocessing may have also influenced the estimated gait-quality measures and should be considered in future work.

## 5. Conclusions

This study demonstrates that gait quality assessed during habitual walking shows measurable age-related differences in healthy adults, with changes particularly evident in stability and step symmetry. Using a single lower-back IMU capturing acceleration and angular velocity, overall gait biomechanics could be evaluated under natural walking conditions beyond traditional performance-based assessments. While physical activity was not associated with gait quality in this cohort, hip extensor strength showed selective relationships with specific biomechanical characteristics, suggesting that hip muscle capacity may contribute to age-related variations in walking. These findings support habitual walking assessments as a feasible approach to studying gait biomechanics in healthy ageing and highlight muscle strength as a relevant factor when interpreting changes in gait quality across adulthood.

## Figures and Tables

**Table 1:** Demographic and clinical characteristics including age, gender, body mass index (BMI), 40 m fast walking performance (40 MFW), the Timed Up and Go test (TUG), stair negotiation, and the 30 s chair stand test (30 SCS). Data are presented as mean (SD) for each age category (20–29, 30–39, 40–49, 50–59, 60–69, and 70–79 years), with 15 participants per group (total *n* = 90). Statistically significant results are highlighted in bold (*p* < 0.05).

*n* = 90	Global Mean	20–29 Years (*n* = 15)	30–39 Years (*n* = 15)	40–49 Years (*n* = 15)	50–59 Years (*n* = 15)	60–69 Years (*n* = 15)	70–79 Years (*n* = 15)	F	*p*	$\omega^{2}$
Age (y)	49.1 (18.4)	21.8 (1.3)	32.8 (2.8)	44.9 (2.9)	55.3 (3.1)	66.3 (2.5)	73.9 (2.6)	-	-	-
Gender (F/M)	54/36	6/9	7/8	10/5	14/1	9/6	7/8	-	-	-
BMI (kg·m^−2^)	24.5 (3.6)	24.1 (3.9)	23.6 (4.1)	25.6 (3.0)	23.9 (3.8)	24.7 (2.7)	25.0 (4.2)	0.69	0.63	−0.00 (−0.00–0.00)
40 MFW	22.01 (3.06)	19.58 (1.76)	20.07 (2.29)	21.35 (3.21)	24.00 (2.98)	22.95 (2.24)	24.12 (2.45)	7.47	**<0.001**	0.11 (0.06–0.21)
TUG	4.37 (1.16)	3.14 (0.46)	3.54 (0.52)	3.61 (0.46)	5.36 (0.75)	5.01 (0.84)	5.57 (0.73)	39.92	**<0.001**	0.47 (0.40–0.57)
Stairs	8.01 (1.99)	6.11 (0.92)	6.43 (0.55)	6.97 (1.02)	9.51 (1.55)	9.11 (1.55)	9.93 (1.70)	23.74	**<0.001**	0.34 (0.27–0.46)
30 SCS	27.74 (6.88)	32.40 (5.73)	29.47 (7.01)	28.00 (5.58)	24.80 (6.91)	25.93 (7.79)	25.87 (6.01)	2.38	**0.05**	0.03 (0.00–0.13)

**Table 2:** Linear regression analyses were performed with age treated as a continuous predictor. Quadratic age terms were only included when they significantly improved model fit. Regression coefficients are presented with standard errors in parentheses. Root mean square error (RMSE) and the coefficient of determination (R^2^) are reported as indicators of model fit. Statistical significance refers to the age coefficient and is indicated by the corresponding *p* value and highlighted in bold.

Gait Parameter	*n*	Intercept	Slope (Age)	Quadratic Term	RMSE	F	*p* (Age)	R^2^
Step symmetry VT	89	0.94 (0.06)	−0.0016 (0.0004)	-	0.07	13.51	**<0.001**	0.12 (0.01–0.30)
Step symmetry ML	89	0.52 (0.08)	−0.0022 (0.0007)	-	0.11	11.26	**<0.01**	0.28 (0.16–0.46)
Step symmetry AP	89	0.79 (0.08)	0.0045 (0.0027)	−0.000063 (0.000028)	0.07	10.36	**<0.001**	0.25 (0.09–0.47)
Stride symmetry VT	89	0.87 (0.06)	−0.0006 (0.0005)	-	0.08	1.24	0.27	−0.01 (−0.03–0.12)
Stride symmetry ML	89	0.45 (0.09)	−0.0008 (0.0007)	-	0.12	1.14	0.29	0.12 (0.02–0.30)
Stride symmetry AP	88	0.79 (0.05)	−0.0006 (0.0004)	-	0.07	2.38	0.13	0.07 (−0.00–0.23)
Stability VT	88	1.05 (0.10)	0.0048 (0.0007)	-	0.12	41.68	**<0.001**	0.32 (0.19–0.50)
Stability ML	89	0.99 (0.11)	0.0036 (0.0008)	-	0.14	17.69	**<0.001**	0.23 (0.11–0.42)
Stability AP	90	1.12 (0.10)	0.0026 (0.0008)	-	0.14	10.25	**<0.01**	0.19 (0.07–0.38)
Smoothness VT	89	−1.67 (0.13)	0.0156 (0.0046)	−0.000160 (0.000046)	0.11	5.97	**<0.01**	0.10 (0.01–0.30)
Smoothness ML	89	−1.40 (0.09)	−0.0001 (0.0007)	-	0.12	0.04	0.83	−0.03 (−0.03–0.09)
Smoothness AP	90	−1.54 (0.14)	0.0015 (0.0011)	-	0.18	1.83	0.18	−0.01 (−0.03–0.15)

**Table 3:** Gait quality outcomes across age groups. Data are presented as mean (SD) for each age category (20–29, 30–39, 40–49, 50–59, 60–69, and 70–79 years), with 15 participants per group (total *n* = 90). Statistically significant results are highlighted in bold (*p* < 0.05).

*n* = 90	Global Mean	20–29 Years (*n* = 15)	30–39 Years (*n* = 15)	40–49 Years (*n* = 15)	50–59 Years (*n* = 15)	60–69 Years (*n* = 15)	70–79 Years (*n* = 15)	F	*p*	$\omega^{2}$
Step symmetry VT	0.83 (0.10)	0.87 (0.07)	0.83 (0.17)	0.85 (0.06)	0.83 (0.07)	0.84 (0.07)	0.78 (0.11)	1.30	0.27	0.01 (−0.01–0.07)
Step symmetry ML	0.61 (0.14)	0.62 (0.11)	0.60 (0.16)	0.65 (0.17)	0.66 (0.13)	0.57 (0.10)	0.55 (0.13)	1.30	0.27	0.01 (−0.01–0.12)
Step symmetry AP	0.80 (0.08)	0.81 (0.06)	0.80 (0.10)	0.83 (0.06)	0.83 (0.04)	0.80 (0.07)	0.73 (0.11)	2.77	**0.02**	0.02 (−0.00–0.10)
Stride symmetry VT	0.83 (0.10)	0.87 (0.06)	0.81 (0.16)	0.83 (0.09)	0.81 (0.10)	0.83 (0.09)	0.84 (0.08)	0.64	0.67	−0.01 (−0.01–0.06)
Stride symmetry ML	0.62 (0.14)	0.65 (0.07)	0.54 (0.17)	0.67 (0.14)	0.65 (0.17)	0.61 (0.14)	0.61 (0.12)	1.02	0.41	0.00 (−0.01–0.09)
Stride symmetry AP	0.77 (0.08)	0.79 (0.06)	0.74 (0.09)	0.79 (0.07)	0.79 (0.07)	0.78 (0.09)	0.74 (0.09)	1.13	0.35	0.00 (−0.01–0.06)
Stability VT	1.25 (0.16)	1.12 (0.16)	1.18 (0.07)	1.26 (0.16)	1.29 (0.13)	1.27 (0.14)	1.38 (0.16)	2.31	**<0.001**	0.02 (0.00–0.09)
Stability ML	1.26 (0.18)	1.23 (0.21)	1.16 (0.16)	1.24 (0.16)	1.36 (0.16)	1.28 (0.19)	1.31 (0.18)	1.79	**0.00**	0.01 (−0.00–0.06)
Stability AP	1.14 (0.15)	1.04 (0.12)	1.11 (0.15)	1.13 (0.13)	1.22 (0.15)	1.14 (0.16)	1.23 (0.14)	1.17	**0.02**	0.00 (−0.01–0.08)
Smoothness VT	−1.34 (0.13)	−1.41 (0.18)	−1.35 (0.11)	−1.28 (0.12)	−1.31 (0.11)	−1.30 (0.08)	−1.40 (0.10)	5.86	0.05	0.07 (0.03–0.17)
Smoothness ML	−1.41 (0.12)	−1.43 (0.15)	−1.42 (0.11)	−1.33 (0.11)	−1.42 (0.09)	−1.42 (0.11)	−1.46 (0.14)	3.70	0.12	0.05 (0.02–0.13)
Smoothness AP	−1.57 (0.18)	−1.63 (0.18)	−1.55 (0.21)	−1.62 (0.16)	−1.53 (0.16)	−1.51 (0.20)	−1.56 (0.16)	2.92	0.33	0.03 (0.00–0.10)

## Data Availability

The data that support the findings of this study are available from the corresponding author upon reasonable request. Processed gait variables and analysis scripts are available for academic use. Due to ethical restrictions, raw participant data containing identifiable information cannot be publicly shared.

## Supplementary Materials

The following supporting information can be downloaded at: https://www.mdpi.com/article/10.3390/s26072194/s1, Table S1. Physical activity across decades derived from International Physical Activity Questionnaire (IPAQ) (N = 90). Table S2. Hip muscle strength characteristics across decades (N = 90).

## Abbreviations

The following abbreviations are used in this manuscript:

IMUs	Inertial measurement units
BMI	Body mass index
IPAQ	International Physical Activity Questionnaire
40MFW	40-m fast-paced walk test
TUG	Time up and go test
30SCS	30 s chair stand test
SaEn	Sample entropy
SPARC	Spectral arc length
VT	Vertical
ML	Mediolateral
AP	Anteroposterior
