# Supplementary material for: Age-Related Differences Across Adulthood in IMU-Derived Gait Quality During Habitual Walking
Source: Sensors (Basel). 2026 Apr 2;26(7):2194. doi: 10.3390/s26072194 (PMC13075253; doi:10.3390/s26072194)
Supplement: Supplementary file 1 [file sensors-26-02194-s001.zip › sensors-4187202-supplementary.pdf]

sensors MDPI

Supplementary material

Table S1. Physical activity across decades derived from International Physical Activity Questionnaire (IPAQ) (N = 90). Global mean and decade-specific values (20–29, 30–39, 40–49, 50–59, 60–69, and 70–79 years; n = 15 per group) are shown for vigorous, moderate, walking, and total activity, expressed as metabolic equivalent minutes per week (MET-min/week). Values are presented as mean (SD).

N=90	Global mean	20-29 years (n =15)	30-39 years (n =15)	40-49 years (n =15)	50-59 years (n =15)	60-69 years (n =15)	70-79 years (n =15)
Vigorous MET	1336 (2359)	1768 (2448)	840 (1809)	886 (1323)	754 (1796)	1671 (2202)	2085 (3773)
Moderate MET	2951 (3532)	1891 (1852)	1579 (1507)	2097 (2083)	4891 (6300)	3743 (3008)	3724 (3640)
Walking MET	1584 (1740)	1749 (1927)	1214 (1973)	779 (738)	1913 (2256)	1840 (1403)	2021 (1631)
Total MET	5872 (5850)	5408 (5289)	3633 (4763)	3762 (2904)	7559 (8922)	7254 (5734)	7830 (5367)

Table S2. Hip muscle strength characteristics across decades (N = 90). Global mean and decade-specific values (20–29, 30–39, 40–49, 50–59, 60–69, and 70–79 years; n = 15 per group) are presented for extensors, flexors, abductors, adductors, internal rotators, and external rotators. Average muscle strength of both limbs (average) and inter-limb difference (diff) are reported as mean (SD).

N=90	Global mean	20-29 years (n =15)	30-39 years (n =15)	40-49 years (n =15)	50-59 years (n =15)	60-69 years (n =15)	70-79 years (n =15)
Extensors_average	169.78 (57.39)	216.54 (46.22)	182.08 (46.05)	203.82 (67.83)	127.59 (17.71)	139.13 (39.24)	130.01 (38.65)
Flexors_average	218.58 (95.84)	306.32 (98.06)	254.78 (66.32)	273.81 (101.86)	139.81 (18.52)	153.54 (40.18)	144.29 (31.21)
Abductors_average	152.46 (38.85)	171.97 (35.08)	163.19 (41.46)	169 (38.34)	129.52 (20.75)	139.96 (41.42)	130.67 (27.83)
Adductors_average	128.64 (45.73)	169 (42.63)	129.75 (38.57)	150.11 (53.32)	92.86 (25.84)	112.22 (27.51)	102.76 (31.97)
Internal rotators_average	134.91 (46.83)	155.56 (45.78)	145.32 (45.88)	170.64 (51.31)	94.55 (10.87)	122.5 (35.53)	104.76 (26.82)
External rotators_average	119.85 (42.21)	148.32 (45.41)	130.09 (33.81)	141.62 (49.83)	83.38 (15.63)	106.55 (27.04)	93.53 (26.94)
Extensors_diff	16.68 (13.21)	17.42 (19.31)	18.14 (10.21)	20.62 (14.28)	15.2 (7.65)	14.27 (11.53)	13.65 (12.38)
Flexors_diff	41.57 (32.54)	22.47 (23.15)	27.81 (29.47)	21.16 (14.36)	63.37 (41.42)	52.19 (25.55)	72.11 (22.21)
Abductors_diff	13 (10.47)	17.79 (12.09)	13.07 (13.86)	12.34 (7.66)	13.28 (7.87)	12.66 (10.14)	8.87 (7.82)
Adductors_diff	11.15 (9.7)	13.4 (14.84)	10.73 (11.92)	14.34 (6.87)	6.49 (5.69)	10.35 (6.72)	9.83 (6.24)
Internal rotators_diff	19.11 (17.83)	22.05 (24.32)	20.17 (14.66)	20.87 (24.96)	13.1 (12.63)	20.11 (14.03)	16.15 (11.5)
External rotators_diff	14.87 (18.97)	22.22 (19.46)	15.89 (18.82)	22.76 (31.88)	6.49 (6.4)	8.7 (9.6)	9.41 (6.62)
